# Supplementary material for: Microglia-mediated demyelination protects against CD8+ T cell-driven axon degeneration in mice carrying PLP defects
Source: Nat Commun. 2023 Oct 30;14:6911. doi: 10.1038/s41467-023-42570-2 (PMC10616105; doi:10.1038/s41467-023-42570-2)
Supplement: Supplementary file 3 — Description of Additional Supplementary Files [file 41467_2023_42570_MOESM3_ESM.pdf]

Description of Additional Supplementary Files

File name: Supplementary Data 1

Description: Complete lists of cluster-specific marker and differentially expressed genes for scRNA-seq data.

File name: Supplementary Data 2

Description: Complete lists of module score genes for scRNA-seq data.
